# Long-Term Glucose Forecasting for Open-Source Automated Insulin Delivery Systems: A Machine Learning Study with Real-World Variability Analysis

**DOI:** 10.3390/healthcare11060779

**Published:** 2023-03-07

**Authors:** Ahtsham Zafar, Dana M. Lewis, Arsalan Shahid

**Affiliations:** 1 School of Electrical Engineering and Computer Science, National University of Sciences and Technology, Islamabad 44000, Pakistan; 2 OpenAPS, Seattle, WA 98101, USA; 3 CeADAR—Ireland’s Centre for Applied AI, University College Dublin, D04 V2N9 Dublin, Ireland

**Keywords:** glucose forecasting, automated insulin delivery, glucose variability, glycemic variability, closed loop, OpenAPS, large-scale diabetes dataset, AID

## Abstract

Glucose forecasting serves as a backbone for several healthcare applications, including real-time insulin dosing in people with diabetes and physical activity optimization. This paper presents a study on the use of machine learning (ML) and deep learning (DL) methods for predicting glucose variability (GV) in individuals with open-source automated insulin delivery systems (AID). A three-stage experimental framework is employed in this work to systematically implement and evaluate ML/DL methods on a large-scale diabetes dataset collected from individuals with open-source AID. The first stage involves data collection, the second stage involves data preparation and exploratory analysis, and the third stage involves developing, fine-tuning, and evaluating ML/DL models. The performance and resource costs of the models are evaluated alongside relative and proportional errors for 17 GV metrics. Evaluation of fine-tuned ML/DL models shows considerable accuracy in glucose forecasting and variability analysis up to 48 h in advance. The average MAE ranges from 2.50 mg/dL for long short-term memory models (LSTM) to 4.94 mg/dL for autoregressive integrated moving average (ARIMA) models, and the RMSE ranges from 3.7 mg/dL for LSTM to 7.67 mg/dL for ARIMA. Model execution time is proportional to the amount of data used for training, with long short-term memory models having the lowest execution time but the highest memory consumption compared to other models. This work successfully incorporates the use of appropriate programming frameworks, concurrency-enhancing tools, and resource and storage cost estimators to encourage the sustainable use of ML/DL in real-world AID systems.

## 1. Introduction

### 1.1. Overview of Data-Driven Automated Insulin Delivery Systems

With an ever-increasing number of diabetes technologies that assist individuals living with insulin-requiring diabetes, large amounts of diabetes-related and user-entered behavioral data are generated. Connected insulin pens or insulin pumps deliver insulin, and real-time blood glucose information is obtained using Bluetooth-enabled glucose meters or continuous glucose monitors (CGM). Insulin pumps and CGM can be combined as part of an automated insulin delivery (AID) system, where data from each device flows through an algorithm to determine insulin-delivery rates and automatically adjust them to keep glucose values in a specific range, requiring less work from people with diabetes and also improving quality of life outcomes [1]. AID systems further generate rich data regarding the conditions (such as sensor glucose values, user-entered information such as targets or carbohydrates, and current and previous insulin delivery) in which it operates [2]. Exploring these rich data sources unveils opportunities for scientific discoveries to understand individual glucose outcomes better and improve diabetes technology.

**Figure 1:**
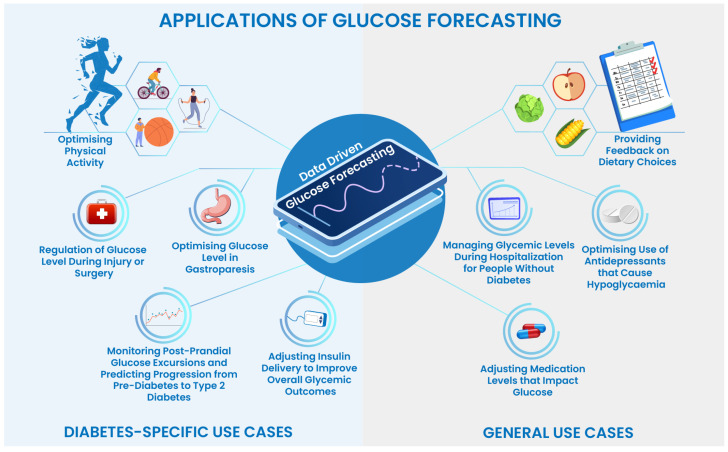
Applications and use cases of data-driven glucose profile forecasting in general healthcare and diabetes-specific scenarios.

There has been increasing interest in applying machine learning (ML) and deep learning (DL) techniques to improve predictions of glucose levels [3]. Accurate and reliable glucose profile forecasting is essential for a range of data-driven applications and use cases that improve diabetes management (Figure 1). ML models are able to train and automatically capture hidden trends and patterns in large volumes of data with considerable accuracy and efficiency. This enables them to make decisions for various prediction and classification tasks and to learn and improve over time.

### 1.2. Applications of Machine Learning and Deep Learning in AID Systems

Several ML techniques, including K-Nearest Neighbour (KNN), Random Forests (RF), Long Short Term Memory (LSTM), Support Vector Regressor (SVR), and Gradient Boost (XGBoost), have been used for regression and classification tasks to predict and identify hypoglycemia and hyperglycemia [4,5,6,7,8,9,10,11,12,13,14]. These methods use invasive and non-invasive techniques to collect data such as continuous glucose monitor data and physiological and demographic features to train the models and achieve high prediction accuracy. Our in-depth review of ML/DL methods applied to glucose forecasting (Section 2.1) yields a list of challenges and limitations to the practical adoption of these methods in open-source AID systems for glucose profile forecasting, including: (1). limited prediction horizon (30, 60, or 120 min) of trained models, (2). inconsistency of reported accuracies and employed model evaluation metrics makes it difficult to compare and reproduce the existing work, (3). unavailability of large-scale and real-world diabetes datasets that encourage the use of artificial and synthetic data for model training and evaluation, (4). lack of evaluation and reporting on the computing resource costs of building the models, (5). lack of implementation details and open-source models that are fine-tuned on diabetes datasets, and (6). lack of assessment of clinically-approved glucose variability metrics (reviewed in Section 2.2) based on predicted glucose profiles.

Historically, due to the non-availability of quality diabetes data, many early datasets used to perform ML-related work were considered “large” if they contained several weeks of data from a dozen individuals. However, with the early adoption of open-source AID systems, which predated the availability of commercial AID systems for several years, users donated their anonymized data for diabetes research [15]. The resulting dataset from the OpenAPS Data Commons contains tens of thousands of days of glucose data points [16] and is employed in this paper.

One unique aspect of open-source AID systems such as OpenAPS is its inherent design to be understandable to users, including the rationale of every decision it makes. ML can be seen as a black box, and it may be challenging to substitute an ML-based prediction algorithm wholesale into an open-source AID. However, OpenAPS is uniquely designed to generate predictions based on various scenarios, including whether carbohydrates are fully absorbed, or a meal is consumed but not recorded to the system. These predictions are conditionally blended and heuristically used [17], such as to produce estimates of the lowest predicted glucose value to be observed over the timeframe relevant for insulin dosing and separately the blended average glucose level over the approximate period when the activity of any additional insulin would be peaking, in order to limit contributions to hypoglycemia while also seeking to minimize hyperglycemia. Therefore, OpenAPS is one such system where an ML-based prediction algorithm could be introduced and blended into the current set of predictions and used alongside the backstop of safety rules used by the system to achieve the highest possible time in the target glucose range (known as “time in range” or TIR) without much hypoglycemia or hyperglycemia.

### 1.3. Original Contributions

As a result of this opportunity for improvement, this paper sought to assess different ML-based prediction methods for glucose profiles, paying particular attention to limitations mentioned above in the existing works [18] and to their performance in terms of accuracy and resource consumption of the implementation (training/inference time and memory consumption) intending to integrate them in open source or future commercial AID solutions.

In this paper, 30 and 60 days of glucose data has been employed from a set of individuals having diverse demographic attributes from OpenAPS Data Commons to train a set of ML and DL models, including ARIMA, XGBoost, RF, SVR, and LSTM. The fine-tuned models have been further evaluated based on their performance and resource consumption for glucose profile prediction up to 48 h. Finally, a set of clinically-validated statistical and glucose variability (GV) metrics have been calculated, and a comparative analysis of the predicted and expected outcomes are presented.

All models have been implemented with the flexibility to train online, and programming scripts are open-sourced for reproducibility and benchmarking [19].

### 1.4. Organisation of the Paper

The rest of this paper is divided into the following sections. Section 2 presents the literature review of tools and technologies for glucose profile assessment and the latest advances in ML-based glucose forecasting methods. Section 3 provides a summary of the dataset and techniques adopted for diabetes data collection, selection and cleaning; followed by a description of employed ML-based predictive models and the glucose analysis metrics. Section 4 presents the glucose variability assessments and the evaluation results of trained ML models for selected individuals with insulin-requiring diabetes. The section further shows the performance and resource costs of ML-based predictive models and reports the relative and proportional errors as a result of a comparison of GV metrics obtained for predicted and expected glucose profiles. Section 5 presents discussions on the analysed ML model outcomes and assessment of metrics used for glucose analysis, highlights the lessons learned, and criticises the limitations. Finally, Section 6 concludes the paper and provides a roadmap for future considerations.

## 2. Related Work

This section first highlights recent research developments towards ML-enabled glucose predictions and highlights the main limitations and challenges; followed by a review of clinically-approved glucose variability metrics.

### 2.1. Review of Machine Learning and Deep Learning Methods and Techniques for Glucose Forecasting

Several machine learning and statistical learning techniques have been employed for regression and classification tasks to predict and identify hypoglycemia and hyperglycemia.

Mordvanyuk et al. [4] employed K-Nearest Neighbour (KNN) algorithm on machine-simulated data and used the meal information along with CGM data to predict out of range glucose with 83.64% accuracy. Dave et al. [5] employed 26 features including gender, the hour of the day, etc as multivariate input in logistic regression (LR) and random forest (RF) algorithms to predict glucose up to 60 min with sensitivity and specificity over 90%. Another approach is the use of physiological data including heart rate and movement recorded by a smartwatch alongside CGM data of an individual employed in the Gradient Boost algorithm to classify normal blood glucose levels and hypoglycemia with an accuracy of 82.7% [6].

Zhu et al. [7] used OhioT1DM dataset [20] to train Long Short Term Memory (LSTM) network to predict up to 30 and 60 min of glucose data and reported root mean square error (RMSE) of 19.10 mg/dL and 32.61 mg/dL, respectively. In [8], simulated data from UVA-Padova [21] (360 simulated days of 10 patients) and OhioT1DM dataset (8 weeks of clinical trials on 6 patients) were employed to train a dilated recurrent neural network (D-RNN) with prediction RMSE of 20.1 mg/dL. Using data from 12 individuals from OhioT1DM, Yang et al. [9] developed an autonomous channel model using a combination of multiple LSTM models for glucose prediction for up to next 30 and 60 min with an RMSE of 18.9 mg/dL and 31.79 mg/dL, respectively.

Berikov et al. [10] used eight CGM-derived metrics including glycemic control and glucose variability from 406 patients in RF, logistic linear regression with lasso regularization, and artificial neural networks (ANN) to predict the next 15 and 30 min of glucose data with considerable accuracy. Duckworth et al. in [11] used explainable ML (trained using CGM data for 153 people with diabetes) to make predictions of hypoglycemia and hyperglycemia up to 60 min. The gradient boost (GB) algorithm yielded a reasonable prediction performance (AUROC) of 0.998 and 0.989 for hypoglycemia and hyperglycemia, respectively, in comparison to standard heuristic and logistic regression models. Van et al. [12] employed a portion of the Maastricht Study’s dataset (including CGM and accelerometer) to train multiple ML and DL models (including ARIMA, support vector regressor (SVR), GB, LSTM, and RNN) and predicted the next 15 and 60 min of blood glucose levels with an RMSE of 0.48 mmol/L and 0.9 mmol/L, respectively. In [13], authors trained a personalized LSTM model (using UVA-Padova simulator data for 100 patients with meals, insulin, and past blood glucose) to predict the next 40 min of blood glucose levels with an RMSE of 7.67 mg/dL.

Allam et al. [14] trained an RNN and SVR using data from 9 individuals to predict blood glucose for 15, 30, and 60 min horizon with an RMSE (in mmol/L) for 0.14, 0.55, 1.32 for RNN and 0.52, 0.89, 1.37 for SVR, respectively. In [22], authors presented an ensemble approach using SVR as a base model and using ARIMA and physiological features (trained on data for 10 individuals with type-1 diabetes) to predict blood glucose levels with RMSE (in mg/dL) of 19.5 and 35.7 for 30 and 60 min prediction horizon, respectively. A jump neural network (JNN) in [23] is trained on data for 20 T1D individuals to predict 30 min of blood glucose with an RMSE (Mean ± Standard deviation) of 16.6 ± 3.1 mg/dL.

Pustozerov et al. [24] trained a linear regression model using data from 62 individuals (with 48 pregnant women with gestational diabetes mellitus (GDM) and 14 women with normal glucose tolerance) with food intake as an evaluation parameter. Results show that the RMSE of BG levels for 1 h after food intake is 0.87 mmol/L. The use of smartwatches has seen tremendous growth with improvements in sensor technology motivated by the use of Photoplethysmography (PPG) signals to detect volumetric changes in blood in the peripheral circulation [25]. Data from 9 people (3 males and 6 females) was used to train ada-boost and RF models to provide 90% prediction accuracy for glucose levels [25]. Dave et al. [5] trained an RF model to predict possible hypoglycemia for 30 and 60 min ahead of time with a sensitivity and specificity of 91% and 90%, respectively.

Georga et al. [26] used multivariate data (including glucose profile, plasma insulin concentration, appeared glucose derived from a meal in the blood circulation, and the energy utilized during other physical activities) from 27 people in free-living conditions in an SVR to predict glucose levels for 15, 30, 60, and 120 min with average prediction errors of 5.21, 6.03, 7.14, and 7.62 mg/dL, respectively. Pérez-Gandía et al. [27] trained a neural network using data from 15 individuals to predict glucose in 15, 30 and 45 min horizon with an RMSE of 10, 18, and 27 mg/dL, respectively.

#### Limitations and Shortcomings

To summarise, multiple ML/DL frameworks and methodologies have been employed to forecast and predict blood glucose for people with diabetes. The limitations and shortcomings of the existing literature are listed below:

- The primary issue of all the reported methods is the evaluation of trained models for a limited prediction horizon of 30 min and 60 min, with the maximum being 120 min, i.e., the reported predictions for the trained models are in the range of 30, 60, or 120 min.
- The lack of consistency in the accuracies of the reported models makes it difficult to compare the existing work. This further affects the reliability of the trained models for further evaluation and reproducibility.
- Another drawback of the existing literature is the previous lack of large-scale and real-world datasets for individuals with diabetes that use automated insulin delivery systems. Therefore, the majority of the aforementioned models in the literature are trained on partial/fully simulated data or limited days of real-world CGM data.
- Multiple model performances and accuracy metrics have been used (including RMSE, specificity, MAE and F1 score) to evaluate the model predictions. However, to the best of our knowledge, none of the existing works has evaluated and studied the impact of glucose predictions by calculating the clinically validated glucose variability (GV) metrics.
- There is a lack of implementation details and open-source methods to reproduce the reported results which makes it difficult to independently evaluate them on additional datasets or to be able to evaluate their applicability for different modalities of insulin therapy, such as in sensor-augmented pump therapy as compared to automated insulin delivery therapy.
- Most of the existing works employed a limited number of machine learning models (one or two) for evaluation which certainly adds inconsistency. However, it is critical to evaluate model results for multiple machine learning and deep learning models along with tuned time series analysis frameworks like ARIMA. Evaluating the results of multiple model types would lay a foundation for benchmarking.

### 2.2. Clinically-Approved Statistical and Variability Metrics for Glucose Analysis

Over 25 clinically approved GV metrics have been adopted by the diabetes research community. Table 1 list the acronyms and full forms of the most important and commonly used metrics for GV assessment.

To assist in the automated calculation and visualisation of clinically approved GV and statistical metrics, many open-source programming tools and frameworks have been developed. These include cgmquantify [28], CGM-GUIDE [29], CGDA [30], EasyGV [31], cgmanalysis [32], and GlyCulator [33].

## 3. Materials and Methods

This section presents the experimental workflow and adopted processes and procedures for diabetes data collection, anonymisation, cleaning, processing, modeling, and analysis.

### 3.1. Experimental Workflow and ML Development Pipelines

**Figure 2:**
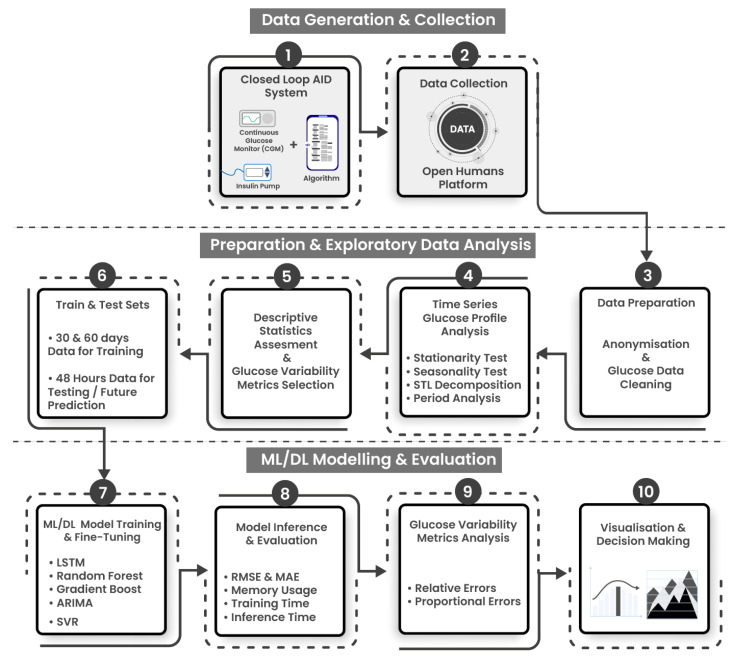
Tri-staged experimental workflow and ML/DL development pipelines for glucose data analysis. Stage 1 includes data generation and collection, stage 2 involves data preparation and exploratory statistical analysis, and stage 3 consists of ML/DL modeling, evaluation and analysis.

The experiments are conducted using a standalone Intel-based Core-i7 CPU processor (2 cores, 2 threads) with 8 GB of main memory. Figure 2 illustrates a tri-staged architecture demonstrating the experimental workflow employed in developing and analyzing ML/DL models.

- Stage 1: Data generation and collection includes data provision from the OpenAPS Data Commons [34], which contains data from open-source AID users who have contributed their data via the Open Humans platform [15] (Steps 1 and 2).
- Stage 2: Data preparation and exploratory data analysis (EDA) is composed of four steps: Data is exported, prepared using anonymization and cleaning protocols (Step 3), a diverse subset of individuals are selected, and the glucose profiles are analyzed using descriptive statistics and clinically approved GV metrics (Steps 4 and 5). The data is then split into training and testing sets. Models have been trained on 30 and 60 days of glucose data and individually tested to predict upto 48 h of glucose data points. (Step 6).
- Stage 3: ML/DL modeling, evaluation and analysis consists of 4 steps. ML/DL algorithms are fine-tuned and evaluated for accuracy and resource consumption (step 7), and analyzed using statistical and glucose variability metrics from expected and predicted glucose profiles (Steps 8, 9, 10).

### 3.2. Highlights of Data Collection, Anonymisation, and Cleaning

The OpenAPS Data Commons, collated as a project on the Open Humans platform, is imported as anonymized diabetes dataset with rich CGM data, insulin delivery information from insulin pumps, user-entered information such as carbohydrate entries or temporary target changes, as well as algorithm-derived information about insulin dosing decisions.

An individual was randomly chosen to test the ML/DL methods described below. After initial tests of methods and validating how much data was needed for analysis, an additional 18 individuals were chosen from the dataset based on the diversity of demographic variables such as ages, AID system used, geography, etc. Table 2 summarizes the demographics of the resulting n = 19 individuals employed in the dataset for this paper, alongside their gender and geography distributions.

Data cleaning methods has been reproduced for timestamps and glucose entries from previous work on glycemic variability [35], and all programming scripts are open-source at [36].

### 3.3. Machine Learning and Deep Learning Algorithms Employed for Glucose Forecasting

Selected ML and DL timeseries forecasting models for glucose include ARIMA [37], XGBoost [38], RF [39], LSTM [40], and SVR [41]. Table 3 provide model descriptions, their fine-tuned hyperparameters for glucose data, and Python implementation library. Although SVR was initially employed to forecast glucose profiles, due to excessive training and execution time and resource consumption, it was dropped and was not considered for further experiments on our dataset. Model evaluation metrics for performance and resource cost are described in Appendix A.

It is important to note that a three-stage process is utilized for ARIMA model building [37]. The first step involves the identification of the order of differencing (d), the order of autoregression (p), and the order of moving average (q) required to model the data. This step involves analyzing the autocorrelation and partial autocorrelation functions of the time series data to determine the values of p and q and analyzing the time series data to determine the value of d. In the second step, parameters have been estimated using maximum likelihood estimation. Lastly, the adequacy of the ARIMA model is checked. This involves analyzing the residuals of the model, which are the differences between the actual data and the model predictions.

When it comes to predicting time series data there are several DL algorithms, however, LSTMs are often considered a reasonable choice for univariate time series prediction due to its ability to handle long-term dependencies and capture temporal patterns in the data. LSTM is a type of recurrent neural network (RNN) that is capable of retaining long-term dependencies in the data, which is particularly useful for time series prediction, where past values can have a strong influence on future values. Unlike traditional RNNs, which can suffer from vanishing or exploding gradients when dealing with long-term dependencies, LSTM has a mechanism to selectively forget or remember information from previous time steps.

Some other conventional DL algorithms were less suitable for our task due to a number of reasons including the inefficiency of univariate time series prediction tasks, computational complexity, and complex hyperparameter tuning. For example, Convolutional Neural Networks (CNNs) are often used for image classification, they can also be applied to time series prediction by treating the time series as a 1D image. However, CNNs may not be suitable for all time series problems, especially if the time series has complex temporal dependencies that cannot be captured by convolutional filters. Similarly, Deep Belief Networks (DBNs) are generative models that consist of multiple layers of Restricted Boltzmann Machines (RBMs) and can be used for unsupervised feature learning. However, they can be computationally expensive to train and may require more data to learn meaningful representations.

### 3.4. Statistical and Variability Metrics for Glucose Analysis

Descriptive statistic metrics are computed for glucose profiles to analyse the spread, variation, and distributions. These metrics include mean, standard deviation (SD), coefficient of variation (CV), skewness score, and quantile statistics (Table 4). Q1, Q2, and Q3 represent the first, second, and third quartiles that evaluate the overall data distribution, respectively. CV indicates the variability in data concerning the mean; the higher the CV is, the more dispersed the data will be. The skewness score is the measure of asymmetric distribution.

A number of clinically approved GV metrics are computed using EasyGV tool [31] and compared for measured (using CGM sensors) and predicted (using ML/DL models) glucose profiles. Relative and proportional errors were calculated and the rationale behind using two error metrics is given in Appendix B.

## 4. Results

This section presents the results of in-depth statistical and GV analysis followed by evaluation and analysis of trained ML/DL models.

### 4.1. Descriptive Statistics and Glucose Variability Metrics for Selected AID Users

Statistical methods are applied to complete glucose profiles for n = 19 individuals to evaluate timeseries data in terms of their characteristics. Stationarity analysis was applied using the augmented Dickey-Fuller (ADF) and Kwiatkowski Phillips Schmidt Shin (KPSS) test. A glucose profile is labeled stationary if both tests conclude that the series is stationary. It is labeled as difference stationary in case only the ADF test is positive and trend stationary if only the KPSS test is positive. It was observed that all the glucose profiles are stationary, with both ADF and KPSS tests being positive. Further analyse was done to evaluate if the time series is seasonal using auto-correlation, and if seasonality is detected, the best period would be found. If the autocorrelation is over 0.9, the data was labelled as seasonal. However, no evident seasonality and periods are detected for selected individuals.

Table 4 reports the descriptive statistics for complete glucose profiles for n = 19 individuals. AID19 had the minimum number of data points (equal to 96 days worth of glucose data), whereas AID3 has the maximum count (constituting 1688 days worth of glucose data). The glucose profile variation is an essential factor in hypoglycemia/hyperglycemia assessment. The minimum and maximum mean values for glucose profiles are 98.42 mg/dL and 158.42 mg/dL, respectively, and the overall average of glucose profiles is 137.56 mg/dL. The minimum, maximum, and average SD for glucose profiles are {30.68, 60.27, 50.15} mg/dL. The average CV for all glucose profiles is 36.36 mg/dL, while the maximum and minimum are 44.37 mg/dL and 26.18 mg/dL, respectively.

Quantiles Q1, Q2, and Q3 determine how many values in a distribution are above or below 25%, 50%, and 75% limits. The minimum, average, and maximum of Q1, Q2, and Q3 are {76, 101.05, 115} mg/dL, {91, 127.94, 153} mg/dL, and {114, 164.78, 195} mg/dL, respectively.

The skewness value greater than ±1 indicates highly skewed distributions. These include AID3, AID7, AID9, AID10, AID15, and AID18. The skewness score between −0.5 and 0.5 (including AID1, AID5, AID8, AID11, AID17, and AID19) indicates symmetrical distributions. The rest of the glucose profiles have skewness scores between 0.5 and 1 or −0.5 and −1, demonstrating that they are moderately skewed.

Table 5 reports the GV metrics. The average SD ROC recorded amidst all glucose profiles is 1.47 mg/dL, whereas the minimum and maximum are 0.79 mg/dL and 2.05 mg/dL, respectively. The minimum and maximum TBR, TIR, and TAR are {0.78%, 16.97%}, {63.6%, 93.9%}, and {2.6%, 32.43%}, respectively. The overall averages for TBR, TIR, and TAR among all glucose profiles are {4.78%, 76.85%, 18.36%}. The recorded average (min–max) for LBGI, HBGI, GMI, and J-Index among selected AID users is 1.23 (0.41–3.82), 4.16 (0.74–6.84), 6.59 (5.66–7.1), and 35.68 (17.35–46.66), respectively.

### 4.2. Performance and Resource Cost Evaluation and Analysis of Trained ML/DL Algorithms

The ML/DL models are trained by employing 30 and 60 days of data and tested individually for their performance and resource costs to predict glucose up to 48 h. Resource costs are evaluated by measuring execution time and memory consumption, whereas RMSE and MAE are calculated to assess the model’s prediction performance.

**Figure 3:**
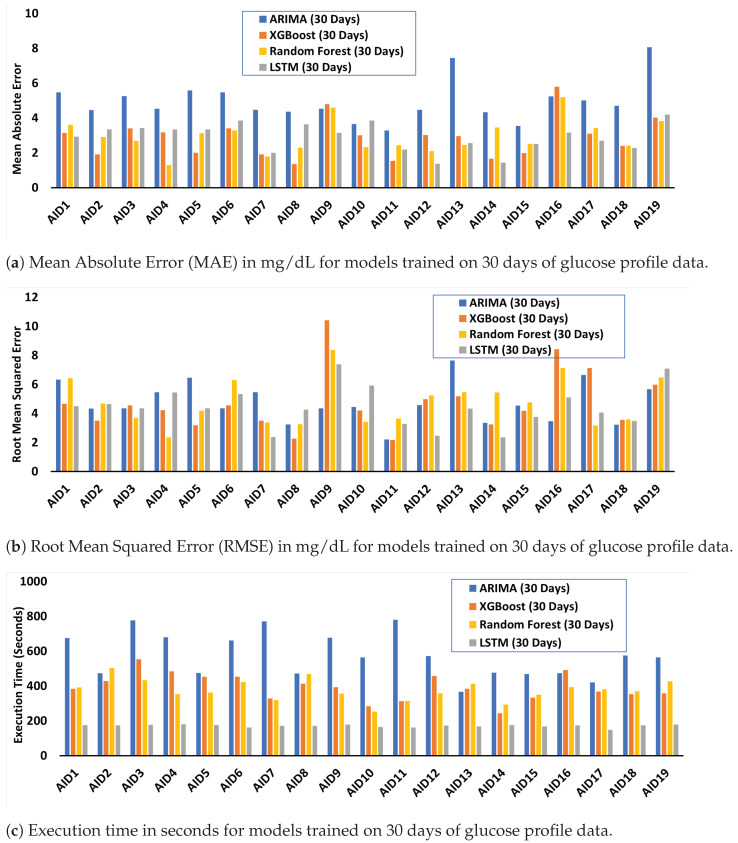
MAE, RMSE, and execution time from ML/DL models employing 30 days of training data.

Figure 3 shows the MAE, RMSE, and execution time for models trained on 30 days of glucose data. The results for models trained on 60 days of glucose data are given in Appendix E.

The maximum value of MAE of 8.07 is observed for ARIMA, whereas the lowest MAE is 1.295 reported for the random forest model (Figure 3a). Overall, the ARIMA model yields the highest MAE indicating the least prediction performance.

The maximum and minimum recorded RMSE is 10.42 for AID9 and 2.16 for AID11, respectively, both in the case of XGBoost (Figure 3b). No noticeable trend was observed between the RMSE values of reported models trained on 30 days of glucose data when compared with the ones trained on 60 days of glucose data.

ARIMA yields a maximum execution time equal to 780 s. In comparison, LSTM performs best in terms of execution time with a minimum of 162 s (Figure 3c. However, LSTMs are recorded as memory-hungry, with consumption peaking at 1993 MBs (Appendix D).

### 4.3. Comparative Analysis of Glucose Variability for Predicted and Expected Glucose Profiles

GV metrics have been calculated from the predicted and expected profiles up to 48 h for n = 19 individuals and evaluate error scores between each GV metric using relative and proportional errors (defined in Appendix B).

Table 6 reports the mean of minimum, average, and maximum relative and proportional errors for GV metrics among selected individuals; obtained by comparing ground truths with the ones calculated using the glucose profiles predicted by ARIMA, XGBoost, LSTM, and RF, respectively. The models trained on 30 days of data are denoted by ARIMA30, XGBoost30, LSTM30, and RF30, respectively. Additional results for the models trained on 60 days of data (ARIMA60, XGBoost60, LSTM60, and RF60) are provided in Appendix F.

Errors have been represented in sets of minimum, average, and maximum. The highest score in the case of ARIMA30 for relative and proportional errors is obtained for TBR with {0%, 11.78%, 54.55%} and {1, 1.12, 1.55}, respectively. The noticeable problem with the relative error is the inconsistency in the maximum error because it considers equal relative proportions for expected and predicted values. Therefore, the proportional error can be considered a comparatively more gaugeable parameter.

The relative and proportional errors obtained by XGBoost30 is the highest for MVALUE equal to {1.67%, 12.18%, 64.69%} and {1.02, 1.12, 1.65}, respectively. For LSTM30, MAG has the highest reported relative and proportional errors equal to {12.54%, 37%, 110%} and {1.14, 1.63, 2.57}, respectively.

The relative errors obtained by RF30 are the highest for MAGE equal to {0%, 18.2%, 182%}. However, the highest proportional errors are obtained for TBR equal to {1, 1.22, 3.5}, respectively.

## 5. Discussion

Large-scale diabetes datasets, such as the OpenAPS Data Commons, provide opportunities for researchers to develop innovative ML/DL tools and technologies and improve the functionality of future automated insulin delivery (AID) systems. This work addresses the limitations of existing ML/DL methods (Section 2.1.1) for predicting glucose profiles by developing models using a dataset of diverse individuals with insulin-requiring diabetes who use open-source AID systems.

ML/DL solutions for diabetes require computing resources, so practical solutions that are fine-tuned and optimized to reduce energy consumption without degrading performance are necessary. This includes using appropriate programming frameworks and tools that enhance concurrency, as well as resource and storage cost estimators and minimizers. Incorporating these strategies ensures the sustainable use of ML technologies and minimizes the environmental impact. In addition to evaluating the accuracy of predictions, it is important to assess the feasibility and sustainability of ML/DL models for use in real-world AID solutions.

The min and max mean values for glucose are likely below average (137.56 mg/dL) due to the use of open-source AID (Table 4). This is confirmed by studies, including a recent RCT [42], which show that open-source AID users typically achieve above-goal glucose metrics. This work also uniquely evaluates data from three open-source AID systems (OpenAPS, AndroidAPS, and Loop). It is worth reflecting that with a decrease in time below range (TBR) and as it is approaching to 0 (which is ideal), the relative error will increase accordingly.

Although AID systems significantly improve glucose management, one should also consider infrequent but significant events such as severe hypoglycemia (a “bad low”) and its long-lasting effects on glucose variability. However, current literature on ML/DL-based glucose forecasting only considers prediction horizons of up to 120 min, hindering the understanding of the relationship between glucose variability and such events. These ML/DL models fine-tuned using the OpenAPS Data Commons accurately forecast glucose profiles up to 48 h (see Appendix C for example profiles). The average MAE range for all trained models is 2.50 mg/dL (for LSTM) to 4.94 mg/dL (for ARIMA). LSTMs have the lowest overall MAE (0.99 mg/dL for AID14) when trained with 60 days of glucose data. The average RSME is 3.7 mg/dL for LSTM to 7.67 mg/dL for ARIMA (Figure 3b).

ML/DL models developed in this work have been evaluated for their computing resource costs. This analysis shows that the execution time of a model is proportional to the amount of data used to train it. For example, models trained on 30 days of data have almost half the execution time of models trained with 60 days of data. LSTMs have the least execution time and the highest memory consumption compared to other models. However, since CPU/GPU time contributes the most to energy-consumption costs, LSTMs are the most resource-efficient in our case. LSTMs could run daily during non-critical times to generate daily predictions, similar to how Autotune, a non-ML-based algorithm for recommending setting changes, runs overnight in OpenAPS [43]. Future work should also consider evaluating cloud computing and the tradeoff costs, including both computing power and the safety risk of off-device calculations in the context of AID.

## 6. Conclusions

Our study comparing GV metrics calculated using predicted and original glucose profiles show the improved accuracy and reliability of extended horizon forecasts in real-world applications. GV metrics are widely used to understand diabetes management outcomes, above and beyond standard glucose outcome metrics, and should continue to be used to evaluate ML/DL-based glucose forecasting methods. The lower error scores in Table 6 show that fine-tuned ML/DL models can accurately estimate glucose variability outcomes for up to 48 h in the future, which is a much longer horizon than has previously been studied with ML/DL methods. Future work should evaluate these methods on different, non-AID diabetes datasets to assess whether ML/DL is “learning” that an AID system will be able to successfully correct according to the forecast; additional work should then also extend this work to assess the utility of such extended forecasts for non-AID users living with diabetes.

The applications of ML/DL described in this paper have the potential to form the basis for intelligent recommender systems in future-generation AIDs and other diabetes applications. In particular, these can be applied thoughtfully to enable individuals to target improvements for their most relevant areas. Quality-of-life improvement could be achieved for people with diabetes by further optimizing exercise, minimizing hypoglycemia, or reducing AID system interaction requirements, all of which can be achieved with future research and applications such as the ML/DL-based forecasts described in this work.

## Figures and Tables

**Table 1:** Clinically Approved Glucose Variability Metrics.

Acronym	Description
ADRR	The average daily risk range (ADRR) measures the overall daily variation of glucose, within a specific risk range meanwhile the risk is defined based on the target.
CONGA	Continuous overall net glycemic action (CONGA) is applicably close to standard deviation (SD) and measures the possible changes in glucose for a defined period.
CV	Coefficient of Variation (CV) is a statistical metric to evaluate the diversity in glucose data and is commonly subdivided into inter-day and intra-day CV metrics.
GRADE	The glycemic risk assessment diabetes equation (GRADE) score evaluates the risk correlated with a particular glucose profile comprehensively.
HBGI	High blood glucose index (HBGI) is a metric that quantifies the possible risk of hyperglycemia and it can be calculated using self-monitoring of blood glucose (SMBG) or continuous glucose monitor (CGM) data.
LBGI	Low blood glucose index (LBGI) is used for hypoglycemic risk management.
MAG	Mean absolute glucose (MAG) represents the difference of summation between sequential glucose profiles over 24 h, which is divided by the time (in hours) between the starting and ending glucose values.
MAGE	The Mean Amplitude of Glycemic Excursion (MAGE) is defined as the mean of glucose values that exceed the 24-h mean blood glucose value, by one standard deviation.
MODD	Mean of daily differences (MODD) evaluates the inter-day variability; the average difference between glucose values is calculated over multiple days at the same time.
SD	Standard deviation (SD) determines the deviation of values in a group from the mean value of the same group of values.
TIR	Time In Range (TIR) quantifies the percentage of time spent within the target sensor glucose range (between 70 mg/dL and 180 mg/dL).
TAR	Time Above Range (TAR) quantifies the percentage of time spent above (>180 mg/dL) the target sensor glucose range.
TBR	Time Below Range (TBR) quantifies the percentage of time spent below (<70 mg/dL) the target sensor glucose range.

**Table 2:** Self-reported demographics of selected AID users within OpenAPS Data Commons.

ID	Age	Daily Insulin (units)	Daily Basal Insulin (units)	Height (cm)	Weight (lb)	Gender	Country	AID Technology
AID1	51–60	52	16.7	195.07	198	Male	Netherlands	OpenAPS
AID2	11–20	66.6	21	155.45	158	Male	Canada	OpenAPS
AID3	21–30	30.97	11.09	170.69	132	Female	Hungary	OpenAPS
AID4	11–20	36.61	9.14	155.45	105	Female	USA	OpenAPS
AID5	11–20	78.63	16.75	155.45	131	Male	USA	OpenAPS
AID6	31–40	86.98	38.72	173.74	277	Female	Canada	OpenAPS
AID7	41–50	45	26	182.88	196	Male	UK	AndroidAPS
AID8	31–40	35	12	164.59	132	Female	Australia	AndroidAPS
AID9	31–40	32	15	167.64	160	Trans Male	USA	OpenAPS
AID10	51–60	44	18	179.83	191	Male	Norway	Loop
AID11	11–20	35	11	167.64	111	Male	Australia	Loop
AID12	41–50	25	14.65	188.98	180	Male	Sweden	AndroidAPS
AID13	11–20	36.31	15.36	146.3	49	Female	Australia	AndroidAPS
AID14	31–40	30	10	167.64	130	Female	USA	OpenAPS
AID15	11–20	35	18	173.74	160	Female	UK	OpenAPS
AID16	21–30	36.85	23.9	179.83	150	Female	Italy	AndroidAPS
AID17	51–60	40	19.5	173.74	224	Female	Germany	OpenAPS
AID18	21–30	90	49	155.75	264	Male	Australia	Loop
AID19	41–50	43.21	26.65	188.98	180	Male	Germany	AndroidAPS

**Table 3:** Machine learning and deep learning model training parameters and their descriptions.

Model	Description	Category	Parameters	Python Library	Optimizing Function
ARIMA	A modelling technique for estimating or foreseeing future results in light of previous time series data. Since constant variance and normal distribution are observed between actual and predicted glucose data, fine-tuned hyperparameters have been reported.	Auto Regressor	P = 7, Q = 0, D = 1, Lags = 7	Statsmodels	ACF, PACF, Stationarity
XGBoost	An additive model is generated by this estimator in a forward fashion which incorporates multiple stages. Further, it adds optimization for differential loss functions. In each stage, a tree is on a negative gradient for a provided loss function.	Regressor	learning rate = 0.1, estimators = 100, sub-sample = 1, max depth = 3	Scikit Learn	Squared Error
Random Forest	A meta assessor that fits various characterizing decision trees on different sub-samples of the dataset and utilizes averaging to work on the exactness and avoid over-fitting.	Regressor	max depth = none, estimators = 100, min sample split = 2	Scikit Learn	Squared Error
LSTM	The models use a progression of ’gates’ to control and manage the data in a string of information as input and output to the framework. There are three gates in a usual LSTM; forget gate, input gate and output gate. These gates can be considered as channels each having its own cognitive framework.	Deep Learning	lags = 1, epochs = 15, batch size = 1, neurons = 50	Keras	Mean Squared Error
SVR	The model implementation is based on libsvm library with high training time complexity, i.e., proportionally more than quadratic with the number of samples. The implementation becomes challenging with large datasets.	Regressor	Kernel = RBF, Gamma = Scale, Epsilon = 0.1, C(regularization param) = 1	Scikit Learn	Epsilon Value

**Table 4:** Descriptive statistics for complete glucose profiles of selected AID users. Abbreviations: Count, count of glucose data points; SD, standard deviation; Q1/Q2/Q3, first/second/third quantile; CV, coefficient of variation.

ID	Count	Mean	SD	Q1	Q2	Q3	CV	Skewness	Distribution
AID1	312,212	147.94	60.27	101	139	185	40.74	0.37	Symmetrical
AID2	357,587	144.51	47.51	110	135	171	32.88	0.93	Moderately Skewed
AID3	486,197	133.09	50.68	96	123	160	38.08	1.08	Highly Skewed
AID4	282,441	140.11	48.22	102	131	169	34.41	0.89	Moderately Skewed
AID5	242,279	145.53	56	106	135	174	38.48	−0.12	Symmetrical
AID6	276,622	140.05	58.85	99	126	167	42.02	0.89	Moderately Skewed
AID7	280,822	127.89	43.35	97	120	151	33.9	1.04	Highly Skewed
AID8	206,778	158.42	57.6	115	153	195	36.36	0.47	Symmetrical
AID9	201,712	116.22	51.57	82	104	137	44.37	1.46	Highly Skewed
AID10	168,848	147.35	55.88	107	135	177	37.92	1.08	Highly Skewed
AID11	163,267	117.18	30.68	95	112	134	26.18	0.30	Symmetrical
AID12	145,692	147.67	53.23	108	138	178	36.04	0.90	Moderately Skewed
AID13	122,557	148.45	55.32	107	138	178	37.27	0.99	Moderately Skewed
AID14	102,673	152.71	56.36	112	138	184	36.91	0.73	Moderately Skewed
AID15	104,669	138.08	40.84	109	130	160	29.58	1.03	Highly Skewed
AID16	96,270	143.14	59.29	101	131	175	41.42	0.98	Moderately Skewed
AID17	77,946	134.24	46.37	100	126	162	34.54	0.13	Symmetrical
AID18	78,798	98.42	33.3	76	91	114	33.84	1.43	Highly Skewed
AID19	27,786	132.75	47.66	97	126	160	35.9	0.45	Symmetrical

**Table 5:** Glucose variability outcomes for complete glucose profiles of selected AID users. Abbreviations: SD ROC, Standard deviation for glucose rate of change; TBR/TIR/TAR, Time before/inside/after range; HBGI/LBGI, High/Low blood glucose index; GMI, Glycemic management index.

ID	SD ROC	TBR (%)	TIR (%)	TAR (%)	LBGI	HBGI	GMI	J_Index
AID1	1.45	6.24	67.13	26.63	1.48	5.85	6.85	43.35
AID2	1.07	1.7	77.77	20.52	0.54	4.42	6.77	36.87
AID3	1.68	5.78	78.05	16.17	1.42	3.64	6.49	33.77
AID4	1.15	1.42	79.1	19.48	0.68	4.12	6.66	35.47
AID5	1.53	4.14	74.33	21.54	0.99	5.1	6.79	40.61
AID6	1.74	4.68	76.29	19.03	1.2	4.77	6.66	39.56
AID7	1.35	4.56	83.76	11.68	1.25	2.71	6.37	29.32
AID8	1.73	3.97	63.6	32.43	0.93	6.84	7.1	46.66
AID9	1.72	14.51	75.06	10.43	3.26	2.56	6.09	28.15
AID10	1.6	2.42	74.36	23.23	0.73	5.31	6.83	41.3
AID11	0.89	2.33	93.9	3.77	0.98	1.23	6.11	21.86
AID12	1.58	2.6	73.55	23.86	0.74	5.19	6.84	40.36
AID13	0.79	2.37	74	23.63	0.73	5.41	6.86	41.52
AID14	2.05	0.78	73.45	25.76	0.43	5.9	6.96	43.71
AID15	1.7	0.83	84.51	14.66	0.41	3.32	6.61	32.01
AID16	1.94	6.13	71.66	22.21	1.48	5.17	6.73	40.98
AID17	1.52	4.3	79.7	16	1.14	3.45	6.52	32.62
AID18	0.95	16.97	80.42	2.6	3.82	0.74	5.66	17.35
AID19	1.59	5.21	79.55	15.24	1.34	3.4	6.49	32.55

**Table 6:** Relative and proportional errors for glucose variability metrics calculated using 48 h of glucose profiles predicted using ARIMA, Gradient Boost, LSTM, and Random forests models employing 30 days of training data.

	Relative Error (%)											
	ARIMA30			XGBoost30			LSTM30			RF30		
	Min	Avg	Max	Min	Avg	Max	Min	Avg	Max	Min	Avg	Max
CONGA	0.12	0.33	0.85	0.02	0.45	1.21	0.01	2.66	44.29	0.06	0.5	1.18
LI	1.86	4.53	10.54	0.24	3.48	11.2	0.06	15.57	207.66	0.02	3.8	15.63
JINDEX	0.02	0.37	0.86	0.03	1.01	3.27	0.03	9.16	163.53	0.06	0.75	2.78
LBGI	0.42	2.85	6.28	0.17	9.84	31.76	0.23	5.91	40.19	0.38	6.07	29.45
HBGI	0.02	1.38	4.52	0.15	3.74	14	0.45	35.39	626.52	0.07	2.87	13.03
GRADE	0.44	2.77	9.78	0.59	4.96	18.24	0.08	20.95	327.02	0.14	4.32	15.47
MODD	0.03	0.68	1.84	0.46	2.18	4.8	0.04	10.59	184.19	0.17	1.46	4.36
MAGE	0.01	6.9	19.88	1.05	10.51	36.59	0.16	9.29	43.58	0	18.2	182.65
ADDR	0.19	3.63	15.73	0.78	4.51	39.98	0.26	47.47	753.86	0.54	5.69	35.9
MVALUE	0.44	3.31	8.65	1.67	12.18	64.69	0.24	8.33	77.16	0.13	7.97	58.22
MAG	6.85	13.68	25.51	0.2	5.29	10.2	12.54	37.11	110.75	12.62	26.48	46
SD	0.14	0.82	1.89	0.53	2.4	5.77	0.28	0.91	2.82	0.07	1.49	4.75
MEAN	0	0.03	0.11	0	0.34	1.06	0.02	0.25	0.77	0	0.27	0.78
CV	0.13	0.81	1.89	0.53	2.55	5.85	0.04	0.91	2.69	0.08	1.59	4.62
TIR	0	0.33	0.91	0	1.04	4.56	0	0.51	2.19	0	1.15	4.12
TAR	0	2.6	14.29	0	1.24	4.44	0	3.68	13.63	0	4.03	13.04
TBR	0	11.78	54.55	0	7.7	40	0	15.76	150	0	11.34	71.43
	**Proportional Error**											
	**ARIMA30**			**XGBoost30**			**LSTM30**			**RF30**		
	**Min**	**Avg**	**Max**	**Min**	**Avg**	**Max**	**Min**	**Avg**	**Max**	**Min**	**Avg**	**Max**
CONGA	1	1	1.01	1	1	1.01	1	1.03	1.44	1	1.01	1.01
LI	1.02	1.05	1.11	1	1.04	1.11	1	1.16	3.08	1	1.04	1.19
JINDEX	1	1	1.01	1	1.01	1.03	1	1.09	2.64	1	1.01	1.03
LBGI	1	1.03	1.06	1	1.1	1.32	1	1.07	1.67	1	1.06	1.29
HBGI	1	1.01	1.05	1	1.04	1.14	1	1.35	7.27	1	1.03	1.13
GRADE	1	1.03	1.1	1.01	1.05	1.18	1	1.21	4.27	1	1.04	1.15
MODD	1	1.01	1.02	1	1.02	1.05	1	1.11	2.84	1	1.01	1.04
MAGE	1	1.07	1.25	1	1.1	1.37	1	1.09	1.44	1	1.17	2.83
ADDR	1	1.04	1.16	1.01	1.05	1.4	1	1.52	8.54	1.01	1.06	1.36
MVALUE	1	1.03	1.09	1.02	1.12	1.65	1	1.09	1.77	1	1.08	1.58
MAG	1.07	1.14	1.26	1	1.06	1.11	1.14	1.63	2.57	1.14	1.38	1.85
SD	1	1.01	1.02	1.01	1.02	1.06	1	1	1.02	1	1.01	1.05
MEAN	1	1	1	1	1	1.01	1	1	1	1	1	1.01
CV	1	1.01	1.02	1.01	1.03	1.06	1	1	1.02	1	1.02	1.05
TIR	1	1	1.01	1	1.01	1.05	1	1	1.02	1	1.01	1.04
TAR	1	1.03	1.14	1	1.01	1.05	1	1.03	1.15	1	1.04	1.15
TBR	1	1.12	1.55	1	1.63	11	1	1.16	2.5	1	1.22	3.5

**Figure A1:**
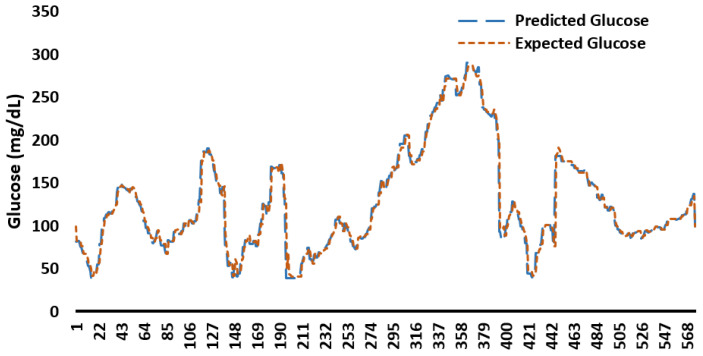
Comparison of expected glucose profiles with predictions from XGBoost trained on 30-day glucose data for 48 h.

**Figure A2:**
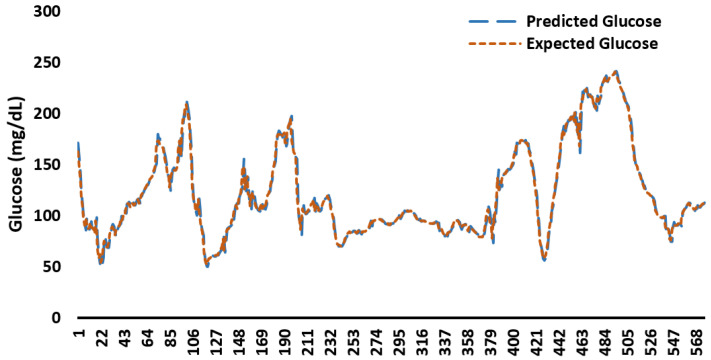
Comparison of expected glucose profiles with predictions from ARIMA trained on 30-day glucose data for 48 h.

**Figure A3:**
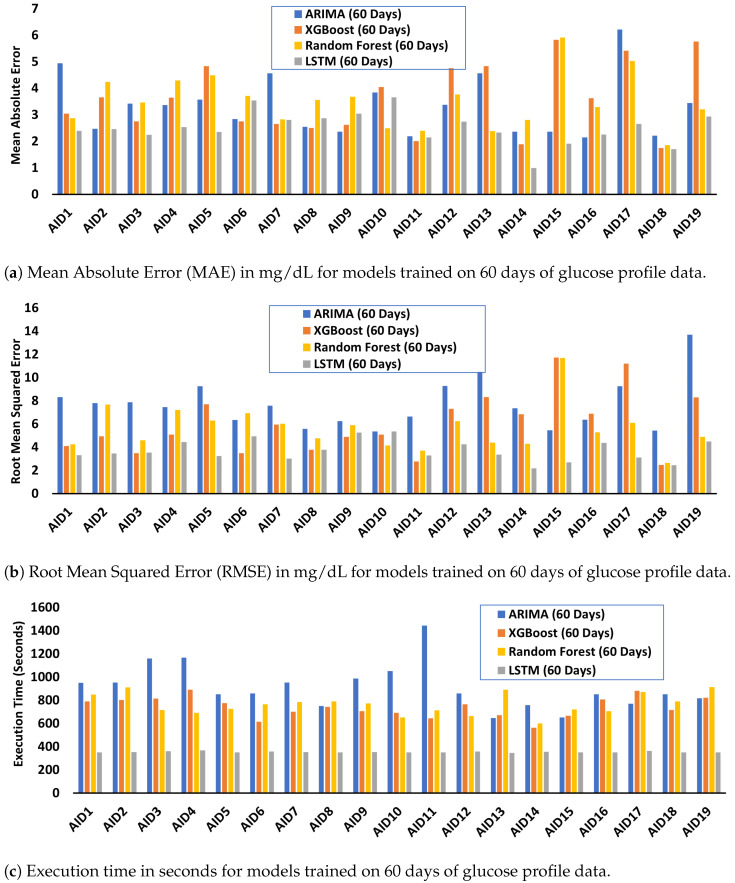
MAE, RMSE, and execution time from ML/DL models employing 60 days of training data.

## Data Availability

All programming scripts and tools developed for the analysis of demographics and glucose data in this paper are made public and online at https://github.com/ahtshamzafar1/ML-and-DL-for-Diabetes-Datasets (accessed on 29 January 2023).

## Abbreviations

The following abbreviations are used in this manuscript:

OPEN	Outcomes of Patients’ Evidence with Novel, Do-it-Yourself Artificial Pancreas Technology
OpenAPS	Open Source Artificial Pancreas System
AID	Automated Insulin Delivery
APS	Artificial Pancreas System
HCL	Hybrid Closed Loop
T1D	Type 1 Diabetes
CGM	Continuous Glucose Monitoring
PWD	People With Diabetes (any type)
HbA1c	Hemoglobin A1c
TIR	Time In Range
GV	Glucose Variability

## Appendix A. Model Evaluation Metrics for Performance and Resource Cost

All the aforementioned models are trained by employing 30 and 60 days of glucose data for each selected individual using closed-loop AID technology and are evaluated for their accuracy and resource costs to predict up to 48 h.

The forecasting accuracy of models is evaluated using Root Mean Square Error (RMSE) and Mean Absolute Error (MAE).

RMSE is calculated as a square root of the second moment of the disparity between expected and predicted data samples and is mathematically defined by Equation (A1); where $\hat{y}$ is the expected value, *y* is the predicted value, and *T* denotes the total number of samples.

(A1) $$\mathrm{RMSE}=\sqrt{\frac{\sum_{t=1}^{T}{\left({\hat{y}}_{t}-y_{t}\right)}^{2}}{T}}$$

MAE provides the average difference between expected and predicted values, whereas the difference between the two is an absolute value. It helps to estimate the disparity between corresponding actual and predicted observations and is mathematically defined by Equation (A2); where $y_{i}$ is the expected value, $x_{i}$ is the predicted value, and *n* denotes the total number of samples.

(A2) $$\mathrm{MAE}=\frac{\sum_{i=1}^{n}\left|y_{i}-x_{i}\right|}{n}$$

Furthermore, in order to assess the suitability of ML and DL models to be employed online and in a real-time application, resource costs have been measured using overall execution time and their memory consumption.

## Appendix B. Relative and Proportional Errors

The relative error (*r*) between a predicted GV metric (*p*) and the expected (ground truth) GV metric (*g*) is given by Equation (A3).

(A3) $$r=\frac{\left|g-p\right|}{g}\times 100\%$$

Relative error (*r*) gives a lower score for a profile that underestimates the GV metric than a profile that overestimates it. This can negatively impact the interpretation of the results. Therefore, proportional error has been further reported.

The proportional error (μ) for predicted GV metric (*p*) with the ground truth (*g*) is a ratio of a maximum of the two values with the minimum of the two values (given by Equation (A4)). The proportional error of 1 indicates no error. The proportional error greater than 1 indicates the difference between the predicted and expected GV metric.

(A4) $$\mu =\frac{max(g,p)}{min(g,p)}$$

## Appendix C. Example Predicted and Expected Glucose Profiles for 48 h

Figure A1 and Figure A2 show the comparison of expected and predicted glucose profiles for 48 h (576 data points) using XGBoost and ARIMA, respectively.

## Appendix D. Memory Consumption by ML/DL Models

**Table A1:** Memory consumption by ML/DL Models during training and testing.

Model	Memory Consumption Range (MB)
ARIMA	994–1024
XG Boost	800–1024
Random Forest	750–1123
LSTM	1100–1993
SVR	1024–1345

## Appendix E. MAE, RMSE, and Execution Time for Models Trained on 60 Days of Glucose Data

Figure A3 shows the MAE, RMSE, and execution time for models trained on 60 days of glucose data. The maximum and minimum reported MAE is 6.21 for the ARIMA and 0.99 for LSTM, respectively (Figure A3a). Figure A3b shows that ARIMA yields the highest error equal to 13.7 for AID19, whereas the minimum RMSE equal to 2.17 for AID14 is obtained for LSTM. Furthermore, LSTM performs best in execution time with a minimum of 346 s.

## Appendix F. Relative and Proportional Errors for Models Trained on 60 Days of Glucose Data

In the case of ARIMA60, the highest relative errors of {0.1%, 45.57%, 705.65%} are obtained for HBGI. However, the highest proportional errors of {1, 4, 50.78} are obtained for ADDR. XGBoost60 yields the highest relative and proportion errors for MAGE equal to {0.18%, 8.76%, 59.67%} and {1, 1.13, 2.48}, respectively. For LSTM60, TBR has the highest reported relative and proportional errors equal to {0%, 51.54%, 700%} and {1, 1.52, 8}, respectively. RF60 yield the highest relative errors for TAR equal to {0%, 13.53%, 100%}. The highest proportional errors for RF60 are reported for MAGE equal to {1, 1.22, 3.38}, respectively.

**Table A2:** Relative and proportional errors for glucose variability metrics calculated using 48 h of glucose profiles predicted using ARIMA, Gradient Boost, LSTM, and Random forests models employing 60 days of training data.

	Relative Error (%)											
	ARIMA60			XGBoost60			LSTM60			RF60		
	Min	Avg	Max	Min	Avg	Max	Min	Avg	Max	Min	Avg	Max
CONGA	0.08	3.85	47.28	0	0.5	1.04	0.1	0.72	2.21	0.13	0.64	1.7
LI	1.11	13.58	88.93	0.01	2.53	6.23	0.48	6.96	12.86	0.57	3.88	15.42
JINDEX	0.04	8.23	79.36	0.06	0.7	1.75	0.12	0.77	2.72	0.07	0.66	2.38
LBGI	0.21	12.27	70.35	2.22	6.05	12.17	0.02	4.52	15.09	0.87	4.39	8.87
HBGI	0.1	45.57	705.65	0.02	1.75	4.32	0.37	4.1	31.74	0.04	7.03	100
GRADE	0.1	20.42	262.81	0.27	4.68	10.2	0.04	2.86	6.45	0	4.59	8.63
MODD	0.06	11.92	152.79	0.31	1.57	2.79	0.33	1.4	5.58	0.03	1.19	2.43
MAGE	1.01	15.52	86.33	0.18	8.76	59.67	0.09	14.75	179.59	0.06	12.54	70.45
ADDR	0.08	43.29	621.83	0.17	1.67	5.35	0.06	8.91	67.25	1.01	12.83	100
MVALUE	0.41	11.6	76.71	1.53	6.38	11.28	0.17	4.07	9.01	0.58	4.15	8.32
MAG	3.52	20.59	100.51	0.14	5.47	11.3	8.63	36.25	60.68	18.18	29.44	45.99
SD	0.17	1.16	4.51	0.02	1.5	3	0.38	1.27	4.02	0.09	0.73	2.15
MEAN	0	0.03	0.13	0.04	0.26	0.82	0	0.4	1.29	0.03	0.34	1.05
CV	0.22	1.17	4.54	0.23	1.57	3.86	0.38	1.47	3.79	0.01	0.7	1.75
TIR	0	0.82	3.16	0	1.77	5.62	0	1.02	5.07	0	1.3	3.84
TAR	0	4.27	21.21	0	3.05	22.22	0	9.25	50	0	13.53	100
TBR	0	25.98	150	0	13.42	50	0	51.54	700	0	11.6	50
	**Proportional Error**											
	**ARIMA60**			**XGBoost60**			**LSTM60**			**RF60**		
	**Min**	**Avg**	**Max**	**Min**	**Avg**	**Max**	**Min**	**Avg**	**Max**	**Min**	**Avg**	**Max**
CONGA	1	1.06	1.9	1	1	1.01	1	1.01	1.02	1	1.01	1.02
LI	1.01	1.4	6.83	1	1.03	1.06	1	1.07	1.14	1.01	1.04	1.18
JINDEX	1	1.24	4.84	1	1.01	1.02	1	1.01	1.03	1	1.01	1.02
LBGI	1	1.21	3.37	1.02	1.06	1.12	1	1.05	1.15	1.01	1.04	1.09
HBGI	1	3.5	40.78	1	1.02	1.04	1	1.04	1.32	1	1.02	1.07
GRADE	1	1.52	7.87	1	1.05	1.1	1	1.03	1.06	1	1.05	1.09
MODD	1	1.15	2.53	1	1.02	1.03	1	1.01	1.06	1	1.01	1.02
MAGE	1.01	1.23	2.86	1	1.13	2.48	1	1.15	2.8	1	1.22	3.38
ADDR	1	4	50.78	1	1.02	1.05	1	1.1	1.67	1.01	1.1	1.47
MVALUE	1	1.25	4.29	1.02	1.06	1.11	1	1.04	1.09	1.01	1.04	1.08
MAG	1.04	1.21	2.01	1	1.06	1.13	1.09	1.69	2.54	1.22	1.45	1.85
SD	1	1.01	1.05	1	1.02	1.03	1	1.01	1.04	1	1.01	1.02
MEAN	1	1	1	1	1	1.01	1	1	1.01	1	1	1.01
CV	1	1.01	1.05	1	1.02	1.04	1	1.01	1.04	1	1.01	1.02
TIR	1	1.01	1.03	1	1.02	1.06	1	1.01	1.05	1	1.01	1.04
TAR	1	1.04	1.21	1	1.04	1.29	1	1.09	1.5	1	1.08	1.5
TBR	1	1.26	2.5	1	1.16	1.86	1	1.52	8	1	1.15	2

## References

[1] Benhamou P.Y., Reznik Y. (2020). Closed-loop insulin delivery: Understanding when and how it is effective. Lancet Digit. Health.

[2] Lewis D.M. (2022). Quantifying input behaviors that influence clinical outcomes in diabetes and other chronic illnesses. J. Diabetes Sci. Technol..

[3] Benhamou P.Y., Franc S., Reznik Y., Thivolet C., Schaepelynck P., Renard E., Guerci B., Chaillous L., Lukas-Croisier C., Jeandidier N. (2019). Closed-loop insulin delivery in adults with type 1 diabetes in real-life conditions: A 12-week multicentre, open-label randomised controlled crossover trial. Lancet Digit. Health.

[4] Mordvanyuk N., Torrent-Fontbona F., López B. Prediction of Glucose Level Conditions from Sequential Data. Proceedings of the CCIA.

[5] Dave D., DeSalvo D.J., Haridas B., McKay S., Shenoy A., Koh C.J., Lawley M., Erraguntla M. (2021). Feature-based machine learning model for real-time hypoglycemia prediction. J. Diabetes Sci. Technol..

[6] Maritsch M., Foll S., Lehmann V., Bérubé C., Kraus M., Feuerriegel S., Kowatsch T., Zuger T., Stettler C., Fleisch E. Towards wearable-based hypoglycemia detection and warning in diabetes. Proceedings of the Extended Abstracts of the 2020 CHI Conference on Human Factors in Computing Systems.

[7] Zhu T., Kuang L., Li K., Zeng J., Herrero P., Georgiou P. Blood Glucose Prediction in Type 1 Diabetes Using Deep Learning on the Edge. Proceedings of the 2021 IEEE International Symposium on Circuits and Systems (ISCAS).

[8] Zhu T., Li K., Chen J., Herrero P., Georgiou P. (2020). Dilated recurrent neural networks for glucose forecasting in type 1 diabetes. J. Healthc. Informatics Res..

[9] Yang T., Yu X., Ma N., Wu R., Li H. (2022). An autonomous channel deep learning framework for blood glucose prediction. Appl. Soft Comput..

[10] Berikov V.B., Kutnenko O.A., Semenova J.F., Klimontov V.V. (2022). Machine Learning Models for Nocturnal Hypoglycemia Prediction in Hospitalized Patients with Type 1 Diabetes. J. Pers. Med..

[11] Duckworth C.J., Guy M.J., Kumaran A., O’Kane A., Ayobi A., Chapman A., Boniface M. (2022). Explainable machine learning for real-time hypoglycaemia and hyperglycaemia prediction and personalised control recommendations. medRxiv.

[12] van Doorn W.P., Foreman Y.D., Schaper N.C., Savelberg H.H., Koster A., van der Kallen C.J., Wesselius A., Schram M.T., Henry R.M., Dagnelie P.C. (2021). Machine learning-based glucose prediction with use of continuous glucose and physical activity monitoring data: The Maastricht Study. PLoS ONE.

[13] Iacono F., Magni L., Toffanin C. Personalized LSTM models for glucose prediction in Type 1 diabetes subjects. Proceedings of the 2022 30th Mediterranean Conference on Control and Automation (MED).

[14] Allam F., Nossai Z., Gomma H., Ibrahim I., Abdelsalam M. (2011). A recurrent neural network approach for predicting glucose concentration in type-1 diabetic patients. Engineering Applications of Neural Networks.

[15] Greshake Tzovaras B., Angrist M., Arvai K., Dulaney M., Estrada-Galiñanes V., Gunderson B., Head T., Lewis D., Nov O., Shaer O. (2019). Open Humans: A platform for participant-centered research and personal data exploration. GigaScience.

[16] Hameed H., Kleinberg S., Doshi-Velez F., Fackler J., Jung K., Kale D., Ranganath R., Wallace B., Wiens J. (2020). Comparing Machine Learning Techniques for Blood Glucose Forecasting Using Free-living and Patient Generated Data. Proceedings of the 5th Machine Learning for Healthcare Conference.

[17] Lal R.A., Maikawa C.L., Lewis D., Baker S.W., Smith A.A., Roth G.A., Gale E.C., Stapleton L.M., Mann J.L., Yu A.C. (2021). Full closed loop open-source algorithm performance comparison in pigs with diabetes. Clin. Transl. Med..

[18] Broome D.T., Hilton C.B., Mehta N. (2020). Policy implications of artificial intelligence and machine learning in diabetes management. Curr. Diabetes Rep..

[19] Zafar A. (2022). Machine Learning/Deep Learning Models and Statistical Analysis Scripts for the Analysis of Glucose Profiles. https://github.com/ahtshamzafar1/ML-and-DL-for-Diabetes-Datasets.

[20] Marling C., Bunescu R. (2020). The OhioT1DM dataset for blood glucose level prediction: Update 2020. Proceedings of the CEUR Workshop Proceedings.

[21] Man C.D., Micheletto F., Lv D., Breton M., Kovatchev B., Cobelli C. (2014). The UVA/PADOVA type 1 diabetes simulator: New features. J. Diabetes Sci. Technol..

[22] Bunescu R., Struble N., Marling C., Shubrook J., Schwartz F. Blood glucose level prediction using physiological models and support vector regression. Proceedings of the 2013 12th International Conference on Machine Learning and Applications.

[23] Zecchin C., Facchinetti A., Sparacino G., Cobelli C. (2014). Jump neural network for online short-time prediction of blood glucose from continuous monitoring sensors and meal information. Comput. Methods Programs Biomed..

[24] Pustozerov E., Popova P., Tkachuk A., Bolotko Y., Yuldashev Z., Grineva E. (2018). Development and evaluation of a mobile personalized blood glucose prediction system for patients with gestational diabetes mellitus. JMIR mHealth uHealth.

[25] Tsai C.W., Li C.H., Lam R.W.K., Li C.K., Ho S. (2019). Diabetes care in motion: Blood glucose estimation using wearable devices. IEEE Consum. Electron. Mag..

[26] Georga E.I., Protopappas V.C., Ardigo D., Marina M., Zavaroni I., Polyzos D., Fotiadis D.I. (2012). Multivariate prediction of subcutaneous glucose concentration in type 1 diabetes patients based on support vector regression. IEEE J. Biomed. Health Inform..

[27] Pérez-Gandía C., Facchinetti A., Sparacino G., Cobelli C., Gómez E., Rigla M., de Leiva A., Hernando M. (2010). Artificial neural network algorithm for online glucose prediction from continuous glucose monitoring. Diabetes Technol. Ther..

[28] Bent B., Henriquez M., Dunn J.P. (2021). Cgmquantify: Python and R Software Packages for Comprehensive Analysis of Interstitial Glucose and Glycemic Variability from Continuous Glucose Monitor Data. IEEE Open J. Eng. Med. Biol..

[29] Rawlings R.A., Shi H., Yuan L.H., Brehm W., Pop-Busui R., Nelson P.W. (2011). Translating Glucose Variability Metrics into the Clinic via C ontinuous G lucose M onitoring: AG raphical U ser I nterface for D iabetes E valuation (CGM-GUIDE©). Diabetes Technol. Ther..

[30] Attaye I., van der Vossen E.W., Mendes Bastos D.N., Nieuwdorp M., Levin E. (2022). Introducing the Continuous Glucose Data Analysis (CGDA) R Package: An Intuitive Package to Analyze Continuous Glucose Monitoring Data. J. Diabetes Sci. Technol..

[31] Moscardó V., Giménez M., Oliver N., Hill N.R. (2020). Updated software for automated assessment of glucose variability and quality of glycemic control in diabetes. Diabetes Technol. Ther..

[32] Vigers T., Chan C.L., Snell-Bergeon J., Bjornstad P., Zeitler P.S., Forlenza G., Pyle L. (2019). cgmanalysis: An R package for descriptive analysis of continuous glucose monitor data. PLoS ONE.

[33] Czerwoniuk D., Fendler W., Walenciak L., Mlynarski W. (2011). GlyCulator: A glycemic variability calculation tool for continuous glucose monitoring data. J. Diabetes Sci. Technol..

[34] OpenAPS Data Commons. https://openaps.org/outcomes/data-commons/.

[35] Shahid A., Lewis D.M. (2022). Large-Scale Data Analysis for Glucose Variability Outcomes with Open-Source Automated Insulin Delivery Systems. Nutrients.

[36] Shahid A. (2022). Programming Scripts for Demographics and Glucose Variability Analysis for OpenAPS Data Commons Dataset. https://github.com/danamlewis/OpenHumansDataTools/tree/master/bin/GV-demographics-scripts.

[37] Newbold P. (1983). ARIMA model building and the time series analysis approach to forecasting. J. Forecast..

[38] Taieb S.B., Hyndman R.J. (2014). A gradient boosting approach to the Kaggle load forecasting competition. Int. J. Forecast..

[39] Masini R.P., Medeiros M.C., Mendes E.F. (2021). Machine learning advances for time series forecasting. J. Econ. Surv..

[40] Siami-Namini S., Tavakoli N., Namin A.S. A comparison of ARIMA and LSTM in forecasting time series. Proceedings of the 2018 17th IEEE international conference on machine learning and applications (ICMLA).

[41] Lin K., Lin Q., Zhou C., Yao J. Time series prediction based on linear regression and SVR. Proceedings of the Third International Conference on Natural Computation (ICNC 2007).

[42] Burnside M.J., Lewis D.M., Crocket H.R., Meier R.A., Williman J.A., Sanders O.J., Jefferies C.A., Faherty A.M., Paul R.G., Lever C.S. (2022). Open-source automated insulin delivery in type 1 diabetes. N. Engl. J. Med..

[43] Lewis D.M., Leibrand S. (2017). Automatic estimation of Basals, ISF, and CARB ratio for sensor-augmented pump and hybrid closed-loop therapy. Proceedings of the Diabetes.

